# Orthotopic liver transplantation in situs inversus adult from an ABO-incompatible donor with situs inversus

**DOI:** 10.1186/1471-230X-14-46

**Published:** 2014-03-13

**Authors:** Songfeng Yu, Hua Guo, Wu Zhang, Jun Yu, Sheng Yan, Jian Wu, Min Zhang, Shusen Zheng

**Affiliations:** 1 Division of Hepatobiliary and Pancreatic Surgery, Department of Surgery, First Affiliated Hospital, School of Medicine, Zhejiang University, Hangzhou 310003, P.R. China; 2 Key Laboratory of Combined Multi-Organ Transplantation, Ministry of Public Health, First Affiliated Hospital, School of Medicine, Zhejiang University, Hangzhou 310003, China; 3 Key Laboratory of Organ Transplantation, Zhejiang Province, Hangzhou 310003, China

**Keywords:** Liver transplantation, Retransplantation, Situs inversus, Abo incompatible

## Abstract

**Background:**

Situs inversus is a rare congenital anomaly characterized by the complete inversion of thoracic and abdominal organs. Liver transplantation in such patients or from donors in situs inversus is technically challenging because of the reversed anatomic structures. A small number of successful liver transplantation cases concerning situs inverus in either recipients or donors have been recently reported with different graft position and orientation. Here we reported an extremely rare case of liver retransplantation from an ABO incompatible situs inversus donor to an adult situs inversus recipient.

**Case presentation:**

A 53-year-old complete situs inversus man developed graft failure due to severe biliary complication after his first liver transplantation from a situs solitus donor. Re-transplantation was performed using a graft liver from a likewise situs inversus donor. Although the blood type between donor and recipient was incompatible, the post-operative outcome was excellent under proper prophylaxis to the antibody-mediated rejection.

**Conclusion:**

To the best of our knowledge, this is the first report of liver transplantation from situs inversus to situs inversus in adult recipient. Liver transplantation using situs matching donor makes the procedure much easier at the surgical point of view, which has a benefit of less potential surgical complications. Furthermore, ABO-incompatibility is acceptable for donor allocation in cases that both donor and recipient are situs inversus.

## Background

Situs inversus (SI) is a rare congenital anomaly characterized by the complete inversion of thoracic and abdominal organs with an estimated incidence in humans about 0.025% to 0.005% live births [1]. The exact etiology of this disorder is currently unknown although choromosomes involved in lateralization and polarity are thought to play an important role [2]. SI may also be associated with other complex anomalies such as biliary atresia, intestinal and vascular malformations [2]. Furthermore, implanting a mirror image liver is technically challenging because of the reversed position and orientation of the graft and hepatic vessels. Thus, SI was previously considered an absolute contraindication for liver transplantation (LTx) until the first LTx in SI patient in 1988 [3]. Since then several cases of successful LTx concerning SI in either recipients or donors have been reported [2,4-12]. However, experiences are still very limited. Liver graft from SI donor still could be discarded from transplantation by some transplant surgeons due to anatomic concerns [13]. The optimal choice for LTx in patients with SI is undoubtedly to receive a similar mirror image liver graft. Given the rare incidence of SI, up to date only one case of LTx from SI to SI was reported in a child receiving a living related graft [14].

Here we report a successful case of LTx from a SI donor to an adult SI recipient. Our case is particular rare and unique because: 1. The patient required re-transplantation due to graft failure after the first LTx from a normal donor; 2. The whole graft from the donor with SI was implanted; 3. The ABO blood type between donor and recipient was incompatible.

## Case presentation

A 53-year-old male was referred to our hospital in September 2012 for re-transplantation due to severe biliary complication after the first LTx. His blood type was “O”. Complete SI without other abnormalities was identified when he was a teenager. Following splenectomy and hepatectomy due to hemorrhage after hepatitis B related cirrhosis and hepatocellular carcinoma respectively, he underwent LTx from a deceased donor with situs solitus because of tumor recurrence in 2005 at the Eastern Hepatobiliary Surgery Hospital, Shanghai, China. Since half a year after LTx, he suffered episodes of biliary complication characterized as jaundice and fever. Bile duct stents were inserted several times. The total bilirubin fluctuated between 70-200 μmol/L and it was gradually elevated to about 400 μmol/L before he was listed for re-transplantation. He had severe skin pruritus and sporadic slight hepatic comma.

**Figure 1:**
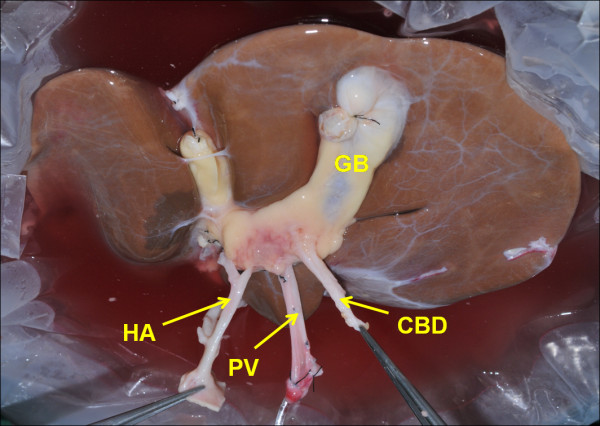
**The liver graft in situs inversus after trimming the excess tissue.** GB: gallbladder; HA: hepatic artery; PV: portal vein; CBD: common bile duct.

The donor was a 32-year-old male who suffered brain injury in a motor vehicle accident and was pronounced dead shortly after admission. Image studies showed that he was situs reversus. His blood type was “B”. Given the organic situs match, the donor liver graft was then allocated to the recipient. No technical difficulties were encountered during the procurement. The liver graft showed completely inverted structure but without vessel anomaly after trimming the excess tissue on back table (Figure 1).

**Figure 2:**
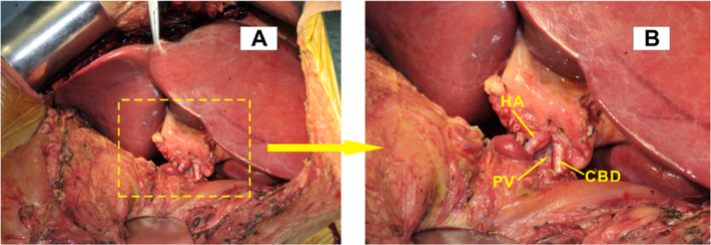
**Intraoperative image after engraftment. (A)** The liver graft fit well in its original orientation and position in the up left quadrant of recipient. **(B)** An amplified view of the portal structure of hepatic artery, portal vein and common bile duct which are in line with and end-to-end anostomosed to the recipient’s corresponding structures. HA: hepatic artery; PV: portal vein; CBD: common bile duct.

After careful laparotomy in recipient, a normal oriented liver was transplanted showing deeply bilious appearance with blunt right lobe and shrunken left lobe. The whole graft was severely rotated to the left upper quadrant. Excision of the original liver graft was performed with preservation of recipient inferior vena cava (IVC). The new liver was then brought onto the field as its original position. The structure of IVC, hepatic artery, portal vein and common bile duct of the graft were in line with the recipient’s corresponding structures. A piggy-back technique with some modifications was applied for cavo-cavostomy. In brief, a longitudinal midline incision on the posterior wall of donor suprahepatic IVC was cut to make a triangulated and wide orifice, which was end-to-side anastomosed to the corresponding similar incision in the anterior wall of recipient IVC from the orifice of hepatic veins. Donor infrahepatic IVC was ligated. The portal vein was end to end anastomosed. The donor’s common hepatic artery was anastomosed to the confluence of the recipient’s proper hepatic artery and gastroduodenal artery. Cholecystectomy was performed as usual and biliary reconstruction was achieved via end-to-end choledochocholedochostomy (Figure 2A, B).

**Figure 3:**
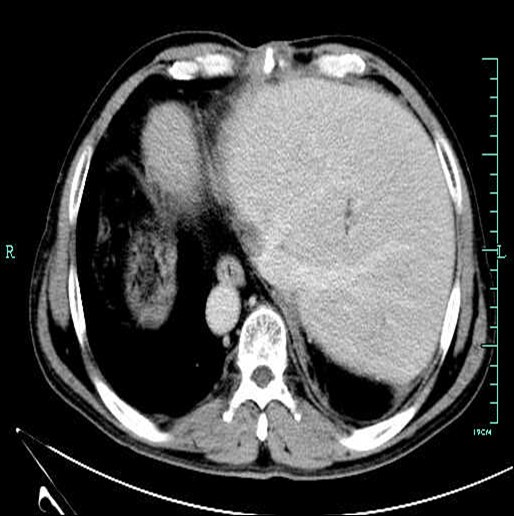
**Graft image surveillance at 3 months post transplant.** Abdominal computed tomography scan shows a normal liver graft in the up left quadrant.

To reduce the risk of complications from ABO mismatch, the patient was treated with the protocol of our center: 600 mg Rituximab (MabThera®, Roche) before transplantation and a total of 200 g intravenous immune globulin during operation and the first ten consecutive days post transplant. The maintenance immunosuppression included tacrolimus, mycophenolate mofetil and steroid. The post-operative outcome was uneventful. He was doing well with excellent graft function at 11 months follow-up. Computed tomography assessment demonstrated the graft in normal appearance in the up left quadrant (Figure 3).

## Discussion

Because of the rare occurrence of SI and the complexity of the situation, there remains limited experiences of LTx in patients with this problem [13]. To date, several different transplant techniques have been described in small number cases of successful LTx from situs solitus to SI or vice versa. In some cases, an orthotopic piggy-back approach was employed with the donor liver in its usual orientation and fashion [4,6,7]. In such cases, graft might shift to lie over the spine for accommodating the recipient’s portal structure. Space occupation by the stomach and spleen and the smaller anatomic graft left lobe might result in the rotation of the graft into the revered “hepatic fossa”. To overcome this problem, Tucker et al. [9] placed a Sengstaken-Blakemore tube to support and immobilize the graft by filling 400 ml saline and gradual balloon deflation. In addition, 90° rotation of the graft could be another alternation for either SI recipient or donor to achieve adequate graft positioning without compromising graft perfusion [8,10,11]. Though the outcomes of these techniques were excellent, potential risk is theoretically associated with distortion and kinking of the anastomosed vessels [5]. More recently, a retroversus implantation technique was reported either in a SI recipient [5] or using a liver graft from donor with SI [12]. This approach flipped the graft 180° along the axis of the IVC (facing backward) and was believed to minimize the risk of torsion, kinking and tension of the anastomosed structures by allowing the liver to sit naturally in an anatomical position in the hepatic fossa without portal structures crossing [5].

In fact, partial LTx from either deceased or living donor has been performed more because such approaches may minimize the difficulties with a smaller graft [15,16]. Nevertheless, only one case of pediatric LTx was reported between familial SI of father and son [14]. The living related left robe with the same mirror-image vessel structure was engrafted, which made the procedure easier and more feasible. In the present case, the patient developed biliary complication shortly after the first LTx. Though no evidence had suggested directly association with anatomic concerns for SI, episodes of biliary complication resulted in chronic graft failure which necessitating a second LTx. Because the present donor was also in the status of situs reversus, the same reversed structure between recipient and donor excluded the orientation and position problem for the engraftment. Thus the risk of distortion and kinking of the anastomosed vessels could be completely avoided. Moreover, there were no more technical difficulties in the operative procedure.

The present case was an ABO-incompatible LTx that was often performed in emergencies when ABO-compatible graft was unavailable. It was used to be a controversial issue because of the high risk of antibody-mediated rejection due to preformed anti-ABO antibodies. Various methods have been applied to improve the outcome of ABO incompatible LTx, including graft local infusion, use of rituximab, intravenous immune globulin, plasma exchange, and splenectomy [17,18]. Especially with the application of rituximab since 2003, a novel anti-CD20 antibody terminating B-lymphocytes, graft survival has been similar to that of ABO-matched transplants. ABO incompatible LTx now has become a feasible option and is no longer an obstacle for liver transplantation [19,20]. From the surgical point of view, situs match makes the transplant procedure much easier and have less potential surgical complications. Although the present case was not in emergency, advantages of the anatomic homomorphism prompted us to perform LTx with ABO-incompatibility between donor and recipient. The patient was treated with an ABO-incompatible protocol of our own using rituximab, intravenous immune globulin. The excellent post transplant outcome and no complications related to blood type mismatch after 6 months follow-up suggested a successful LTx.

## Conclusion

The current case is the first report in adult LTx from SI donor to SI recipient. Form the surgical point of view, the same mirror-image liver vessel structure makes the present procedure much easier and thus have less potential surgical complications. Given the limited experiences of LTx in SI, if situs matches, we suggest that it’s better to allocate SI donor to SI recipient. ABO-incompatibility is acceptable for donor allocation in cases that both donor and recipient are SI.

## Consent

Written informed consents were obtained from both the recipient of the transplant and from the next-of-kin of the donor for publication of this case report and all accompanying images. The copies of the written consent are available for review by the Editor-in-Chief of this journal.

## Abbreviations

SI: Situs inversus; LTx: Liver transplantation; IVC: Inferior vena cava; GB: Gallbladder; HA: Hepatic artery; PV: Portal vein; CBD: Common bile duct.

## Competing interests

The authors declare that they have no competing interests.

## Authors’ contributions

YS reviewed the data and literature and wrote the manuscript. GH collected and reviewed the literature. ZW and YJ participated in the donor procurement. YS, ZM and SY participated in the operation and the treatment of recipient. ZS contributed to revising the manuscript. All authors have read and approved the final manuscript.

## Pre-publication history

The pre-publication history for this paper can be accessed here:

http://www.biomedcentral.com/1471-230X/14/46/prepub
